# Skin-to-skin transfer from the delivery room to the neonatal unit for neonates of 1,500g or above: a feasibility and safety study

**DOI:** 10.3389/fped.2024.1379763

**Published:** 2024-03-20

**Authors:** Meline M’Rini, Loïc De Doncker, Emilie Huet, Céline Rochez, Dorottya Kelen

**Affiliations:** Neonatal Department, Hôpital Universitaire de Bruxelles, Hôpital Erasme, Université Libre de Bruxelles, Brussels, Belgium

**Keywords:** skin-to-skin, transfer, transport, neonates, low-birth-weight, respiratory support, safety, feasibility

## Abstract

**Objective:**

Immediate skin-to-skin contact (SSC) is already standard care for healthy term newborns, but its use for term or preterm newborns requiring admission to neonatal intensive care unit (NICU) with or without respiratory support is challenging. This study aimed to assess the safety and feasibility of SSC during the transfer of newborn infants, using a new purpose-built mobile shuttle care-station, called “Tandem”.

**Material and methods:**

A monocentric prospective observational study was conducted at the tertiary referral center of the Université libre de Bruxelles in Brussels, Belgium after ethical approval by Hopital Erasme's Ethics Committee (ClinicalTrials.gov ID: NCT06198478). Infants born with a birth weight above 1,500 g were included. Following initial stabilization, infants were placed in SSC with one of their parents and transferred to the NICU using the Tandem.

**Results:**

Out of 65 infants initially included, 64 (98.5%) were successfully transported via SSC using the Tandem. One transfer was not successful due to last minute parental consent withdrawal. The median (range) duration of continuous skin-to-skin contact after birth was 120 min (10–360). SSC transfers were associated with gradually decreasing heart rate (HR) values, stable oxygen saturation levels (SpO_2_), and no increase in median fraction of inspired oxygen (FiO_2_). Heatloss was predominantly observed during initial setup of SSC. There was no significant difference in the occurrence of tachycardia, desaturation or hypothermia between preterm and term neonates. No equipment failures compromising the transfer were recorded.

**Conclusion:**

Skin-to-skin transfer of infants with a birthweight of equal or above 1,500 g using the Tandem shuttle is feasible and associated with stable physiological parameters. This method facilitates early bonding and satisfies parents.

**Clinical Trial Registration:**

ClinicalTrials.gov (NCT06198478).

## Introduction

Prematurity, defined as birth occurring before 37 weeks of gestation, remains a significant global health concern. Preterm birth affects approximately 10% of all births worldwide, making it the leading cause of neonatal mortality and the second leading cause of mortality among children aged 1–5 years old (1–3).

Neonatal intensive care unit (NICU) admission rates have increased over the years. Therefore, effective management and care for premature newborns are crucial in reducing overall child mortality rates. A substantial proportion of NICU admissions consists of premature or term infants above 32 weeks of gestation or with a birth weight of above 1,500 g. These infants continue to undergo critical brain development, while being subject to parental separation, therefore creating an optimal environment for their neurodevelopment while keeping them close to their parents is a primary NICU objective.

Early mother-infant separation became common in the 20th century with the shift from home births towards hospital deliveries. However, this practice is not aligned with current findings that emphasize the critical role of close and continuous mother-infant skin-to-skin contact (SSC). SSC involves placing the undressed newborn directly on the bare chest of their primary caregiver. The benefits of SSC during hospitalization of preterm infants include improved thermoregulation, cardiopulmonary stability, longer duration of exclusive breastfeeding, reduced stress levels in both infants and caregivers, minimized procedural pain, improved bonding, enhanced long-term neurodevelopmental outcomes, and regulated sleep-wake patterns (4–8).

In accordance with recent WHO recommendations, SSC should be initiated as soon as possible after birth for preterm or low-birth-weight infants and should be incorporated as a routine care practice as much as possible (3). While Belgian maternity services actively promote SSC in healthy full-term infants, the standard NICU procedure for admissions involves the use of a transport incubator, separating newborns from their parents.

Maintaining immediate and continuous SSC during NICU transfers from the delivery room is challenging but essential to prevent separation.

To facilitate SSC for parents and newborns during NICU transfers, a mobile care station attached to a wheel chair or to the bed of the mother called the “Tandem,” was designed. This study aims to assess the feasibility and safety of SSC during NICU transfers using the Tandem device for neonates with a birth weight of 1,500 g or above.

## Materials and method

### Study design and ethics

This monocentric prospective observational study was conducted at Hopital Erasme from March 2017 to December 2019. Ethical approval was obtained from Hopital Erasme's Ethics Committee (Approval ID: P2019/510). It was also registered at ClinicalTrials.gov (ID: NCT06198478).

### Study population

Neonates with a birth weight of 1,500 g or above were eligible for inclusion to the study. Informed consent was obtained from parents before delivery. Neonates were enrolled if a trained research team member was present and at least one parent was available for SSC transfer. Exclusion criteria comprised severe congenital malformations contraindicating SSC, the need for invasive ventilation, or parental refusal.

### Equipment

**Figure 1:**
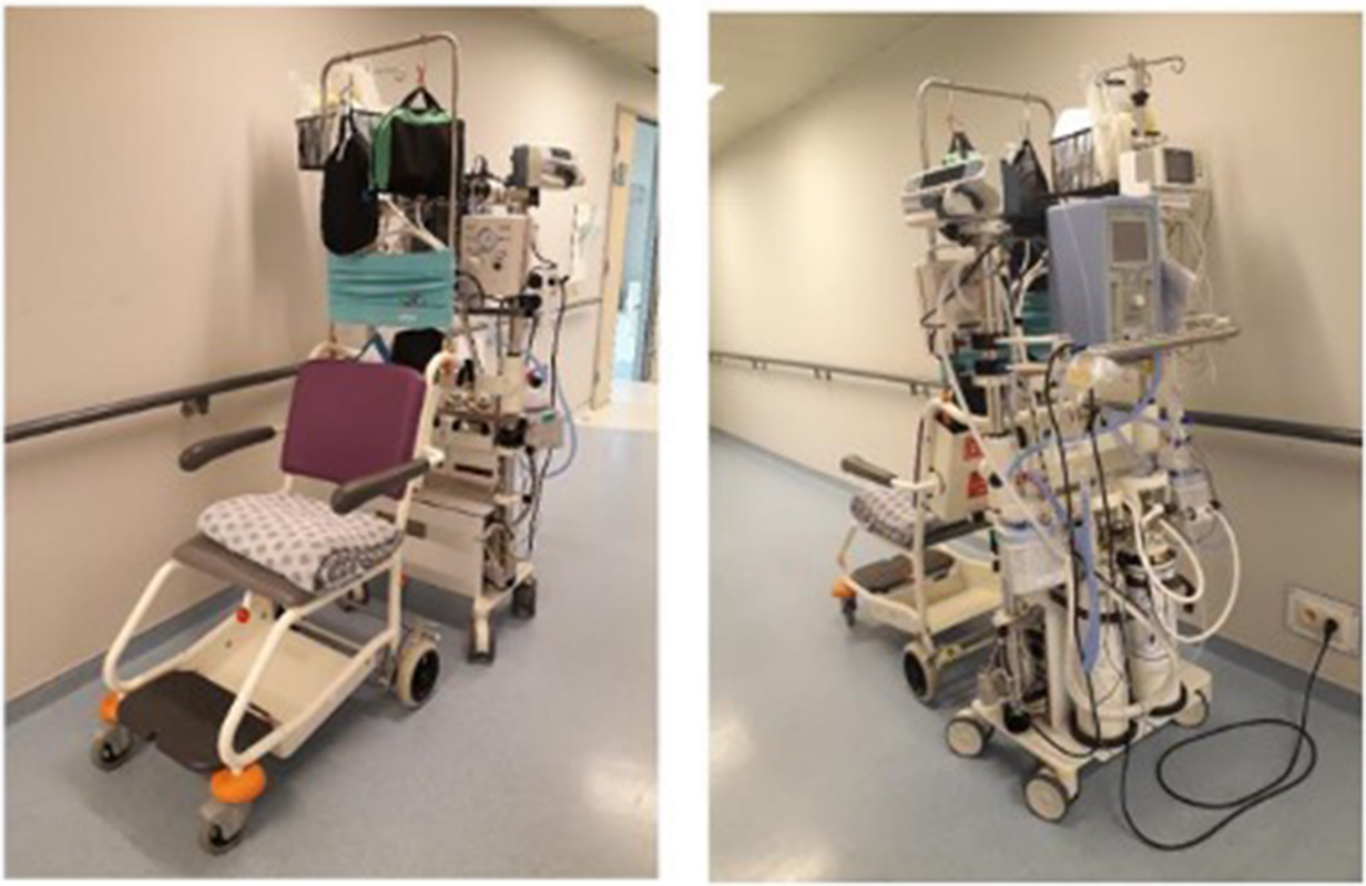
Picture of the Tandem.

The Tandem is a mobile care station which can be attached to a wheel chair or the bed of the mother. It is equipped to provide respiratory support and continuous monitoring, and enables intravenous administration of fluids or drugs, allowing a care-holder to sit or lie while maintaining SSC with their infant (Figure 1).

The equipment includes:

- Neopuff ventilator with tubing and different mask sizes
- Non-invasive ventilators: nasal continuous positive airway pressure (CPAP), high-flow nasal cannula (HFNC) and their respective humidifiers
- Continuous monitoring of heart rate, saturation, fraction of inspired oxygen and temperature with ECG, saturation and temperature probes
- Nasogastric tubes
- Intravenous (IV) catheters and materials for drug administration or infusions
- Batteries
- Miscellaneous accessories such as a holding tube top designed to be worn around their chest by the parent and in which the neonates are held, hats, and covers

### Procedure

Prior to delivery, the trained neonatal team prepared both the Tandem and a transport incubator as a backup. Newborns were delivered either vaginally in the delivery room or via caesarian section in the operating room.

According to Hopital Erasme's local protocol, delayed umbilical cord clamping was performed for at least one minute unless prompt resuscitation was required, in which case immediate cord clamping was implemented. Then, infants were stabilized on a resuscitation table following European Resuscitation Council guidelines (9). Oxygen saturation (SpO_2_) and electrocardiography probes were applied (right arm for preductal saturation and on the infant's chest respectively).

After initial stabilization and based on the attending clinician's decision, the type of respiratory support needed was initiated using the Tandem's gas supply. In cases when antibiotics or continuous IV glucose infusion was necessary, peripheral venous catheters were placed before departure. A nasogastric tube was also inserted if needed, based on the attending clinician's decision. After parents were comfortably accommodated in their beds or chairs, undressed neonates were placed against their bare chest secured by a tube-shaped holding top, and the parent-infant duo was covered by two layers of additional blankets. Finally, continuous skin temperature monitoring via a dorsal temperature probe was also started.

Infants were then transferred to the NICU under the supervision of a pediatric nurse and/or pediatrician. Whenever possible, SSC was continued for at least 120 min before placing the infant in an incubator.

### Primary outcome and safety parameters

The safety and feasibility of the SSC transfer procedure was assessed by continuous monitoring of hemodynamic, respiratory and thermal parameters that were recorded at defined timepoints: during installation of SSC, at the start of the transfer, upon NICU arrival, and after 60 and 120 min in the NICU. Blood glucose levels were measured at least once during the procedure.

To further evaluate the safety of the procedure, incidences of adverse events were also recorded. The normal values were set according to local protocol and scientific literature: we considered heart rates (HR) below 120 bpm or above 180 bpm for more than 4 s, SpO_2_ below 92% for more than 50 s, temperature below 36.5°C or above 37.5°C, and a blood sugar of below 30 mg/dl respectively abnormal.

### Secondary outcomes

The occurrence of technical issues and/or equipment failure such as issues with the wheelchair, the oxygen saturation or electrocardiography probes, the monitor or the ventilators as well as any dislodgement of any medical device placed (nasal cannula, IV catheter, nasogastric tube) which could compromise the SSC transfers were recorded. Parental and nursing satisfaction with SSC transfer via the Tandem from the delivery suite or the operating theater to the NICU were rated on a scale from 1 to 10.

### Statistical analysis

Data were exported anonymously, and analysis was conducted using GraphPad Prism version 10.0.2 for macOS, GraphPad Software. Categorical variables were summarized as frequencies and percentages, while continuous variables were presented as medians with ranges or interquartile ranges. Statistical significance between groups was assessed using Pearson's chi-square test or Fisher's exact test for categorical variables when appropriate, with a two-sided significance level of 5% (*p* < 0.05).

## Results

**Figure 2:**
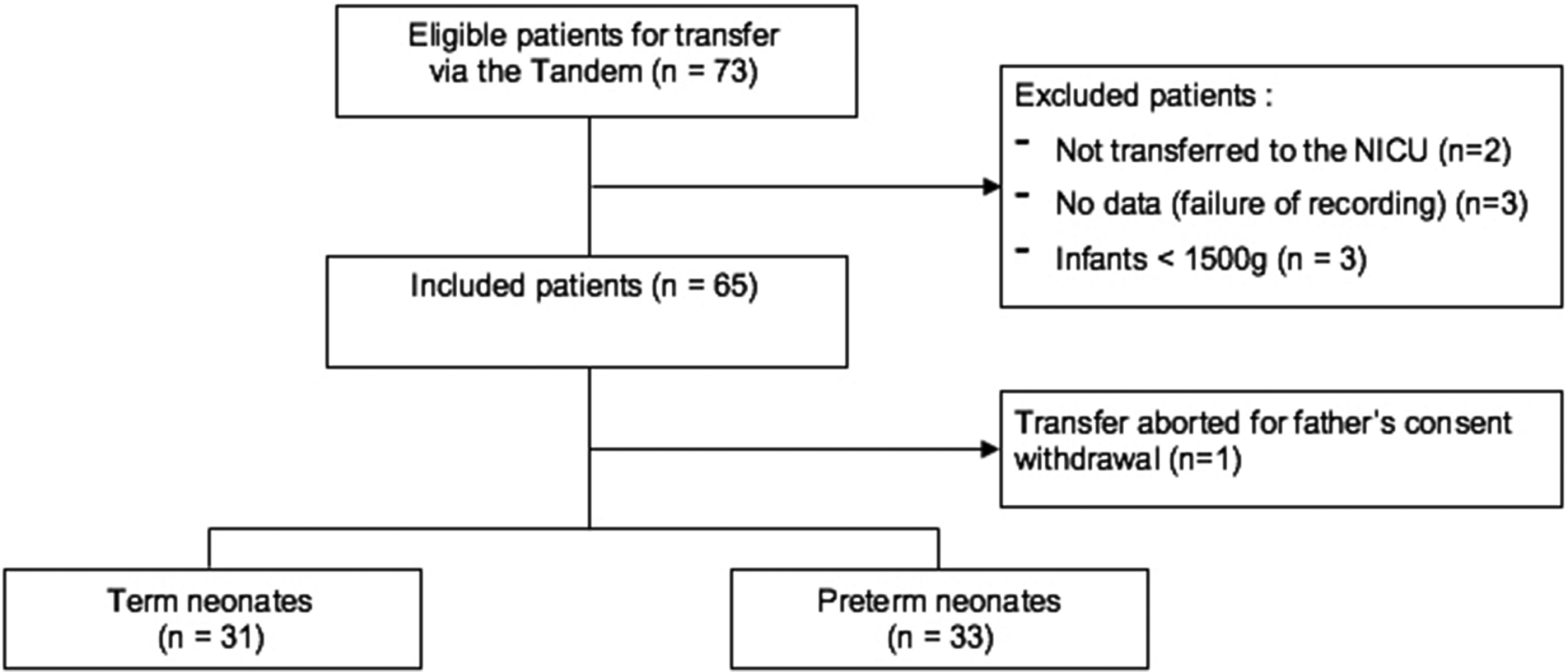
Recruited patients and cohort population.

Between March 2017 and December 2019, a total of 73 neonates were eligible for the study. Of these, two neonates were excluded due to transfer via the Tandem from the delivery room to another destination (to the operating room for immediate surgical care). Additionally, three neonates were excluded due to failure of data recording, and three more were excluded for having a birth weight below 1,500 g upon weighing after the end of the procedure. Ultimately, 65 neonates with a birth weight of 1,500 g or above were included in the study, 31 term and 33 preterm neonates. One transfer was deemed unsuccessful due to last-minute parental consent withdrawal (Figure 2).

The cohort's baseline characteristics can be found in Table 1.

**Table 1:** Baseline characteristics.

Characteristics	Preterm neonates <37 weeks (*n* = 33)	Term neonates ≥ 37 weeks (*n* = 31)
Delivery		
Cesarean section	11 (33.3%)	14 (45.2%)
Vaginal delivery	22 (66.7%)	17 (54.8%)
Gestational age (weeks)	31 1/7–36 4/7	37 0/7–42 0/7
Birth weight (grams)	2,135 (1,700–3,060)	3,370 (1,745–4,540)

Data are presented as median (range) and *n* (%).

Concerning perinatal outcome, median Apgar scores were 9-9-10 and 8-9-9 at 1, 5 and 10 min for preterm and term neonates, respectively. Respiratory support was required for 39.4% (CPAP 21.2%; HFNC 18.2%) and 74.2% (CPAP 29.0%; HFNC 45.2%) of preterm and term neonates, respectively (Table 2). Late cord clamping was performed for most of the neonates (median = 2 min).

**Table 2:** Perinatal outcome.

	Preterm neonates (*n* = 33)	Term neonates (*n* = 31)
Apgar [median (min—max)]		
Apgar 1′	9 (3–10)	8 (1–10)
Apgar 5′	9 (7–10)	9 (4–10)
Apgar 10′	10 (8–10)	9 (8–10)
Respiratory support		
None	20 (60.6%)	8 (25.8%)
CPAP	7 (21.2%)	9 (29.0%)
HFNC	6 (18.2%)	14 (45.2%)

Data are presented as median (range) and *n* (%).

CPAP, continuous positive airway pressure; HFNC, high-flow nasal canula.

The moment of SSC initiation ranged from 0 min (neonate immediately placed on their mother) to 75 min, however most of the neonates benefited from early SSC (median = 15 min). Median time of SSC was 120 min up to 360 min. Most of the transfers were performed with the father (81%). Nasogastric tube and IV access were placed in 53.1% and 35.9% of neonates, respectively (Table 3).

**Table 3:** SSC transfer characteristics.

	*n* (%)	Median (min—max)
Cord clamping (min) Unknown = 5		2 (0–2)
Start of SSC (min after birth) Unknown = 1		15 (0–75)
Duration of SSC (min) Unknown = 6		120 (10–360)
Parent performing SSC Unknown = 1		
Mother	12 (19%)	
Father/birthing partner	51 (81%)	
Nasogastric tube	34 (53.1%)	
Intra-venous access	23 (35.9%)	

Data are presented as median (range) and n(%).

SSC, skin to skin contact; CPAP, continuous positive airway pressure; HFNC, high-flow nasal canula.

**Figure 3:**
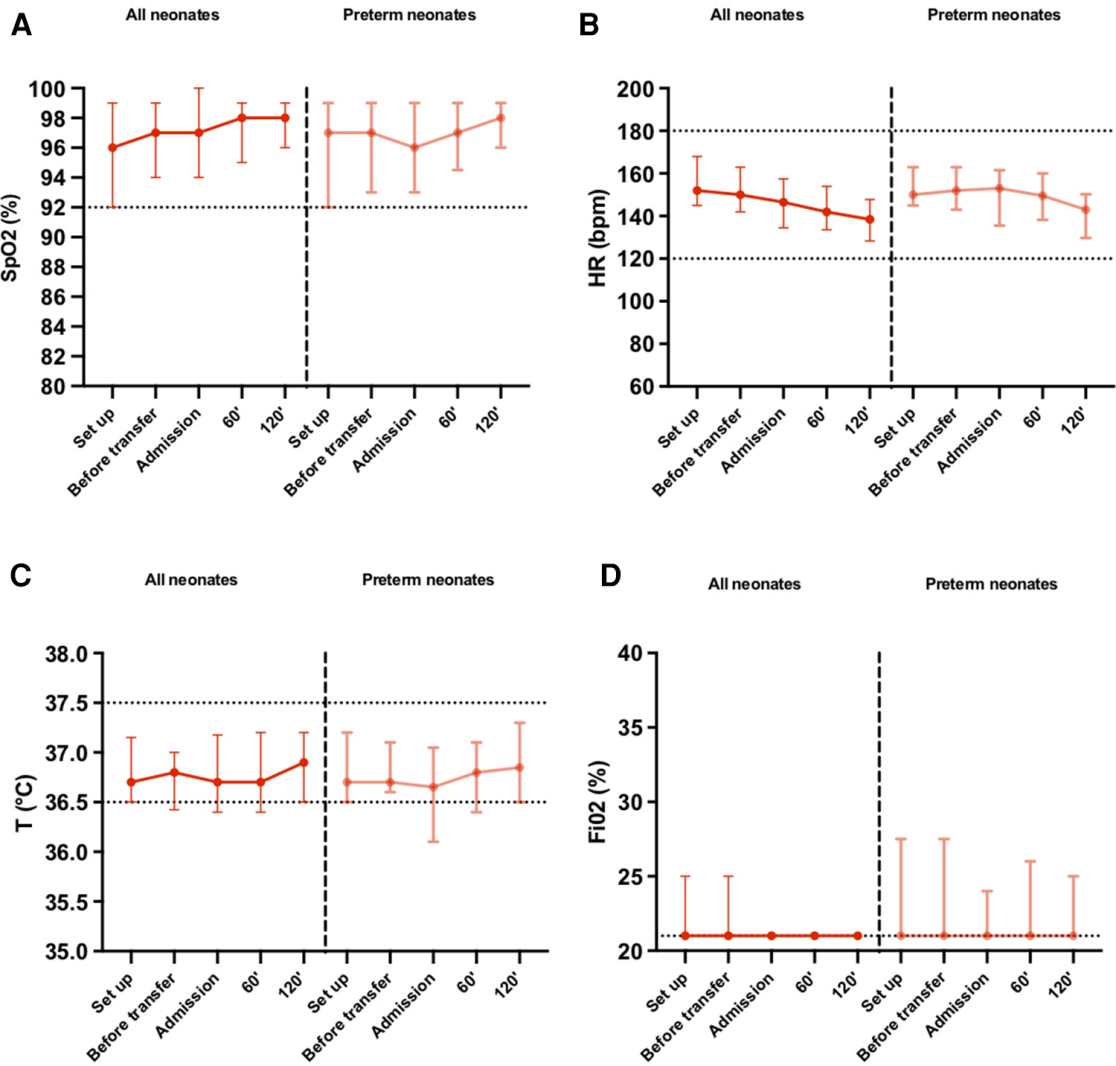
(**A**) Oxygen saturation, (**B**) heart rate, (**C**) cutaneous temperature, (**D**) fraction of inspired oxygen, recorded at defined time points until 120 min after arriving at the NICU. Parameters are presented as median (IQR) for all neonates and neonates born preterm (<37 weeks gestational age). SpO_2_, oxygen saturation; HR, heartrate; T°, temperature (°C); FiO2, fraction of inspired oxygen.

The median oxygen saturations, heart rates and temperatures were maintained within normal range during the procedure. Overall for all neonates, the median heart rate value gradually decreased after birth. For preterm neonates, the median heart rate increased slightly until NICU admission while staying within normal range, then decreased progressively (Figure 3).

For 8 (12.7%) neonates, heart rate value was measured above 180 bpm at least once during the procedure. Five (7.9%) of the neonates had a heart rate above 180 bpm only at installation, 2 (3.2%) neonates continued to have a heart rate value above 180 bpm until before departure and one (1.6%) of them had a heart rate still above 180 bpm 2 h after arriving in NICU. The latter was born at term by emergency caesarian section due to fetal bradycardia. Oxygen saturation was measured below 92% for 17 (27%) neonates at least once during the procedure. There were 10 (18.9%) neonates who had their SpO_2_ below 92% during installation, and 6 (11.5%) before departure. Upon arrival, 3 (6.5%) had a saturation below 92%. 14 (22.2%) neonates, including 7 preterm, required at some point an increase in the fraction of inspired oxygen during the procedure. The maximum FiO_2_ required was of 50%, which was for one neonate (1.6%) and was only during the initial setup then was progressively decreased to 21% during the procedure.

Heart rate and oxygen saturation was unknown for one patient.

The cutaneous temperature was measured below 36.5°C for 27 (42.2%) neonates, and below 36°C for 10 (15.6%) neonates at least once during the procedure. There were 10 (20.4%) neonates who had a temperature below 36.5°C during installation, and 5 (10.2%) with a temperature below 36°C. Before the departure, 11 (26.8%) had a temperature below 36.5°C and only 3 (7.3%) of those had a temperature below 36°C. Upon arrival, 14 (31.1%) had a temperature below 36.5°C and 4 (8.9%) of those had a temperature below 36°C. At 120 min, 7 (15.2%) had a temperature below 36.5°C and only 1 (2.2%) of those had a temperature below 36°C. The lowest temperature recorded was of 35.1°C (*n* = 1, 1.6%), followed by 35.5°C (*n* = 1, 1.6%).

There was no significant difference in the occurrence of tachycardia (HR above 180 bpm), desaturation (SpO_2_ below 92%) or hypothermia (T°C below 36.5°C) between preterm and term neonates (Table 4).

**Table 4:** Occurrence of adverse events in term and preterm neonates.

	Preterm neonates (*n* = 33) *n* (%)	Term neonates (*n* = 31) *n* (%)	*P*-value
Tachycardia Unknown	4 (12.1%)	4 (13.3%) 1	1
Bradycardia Unknown	3 (9.1%)	3 (10%) 1	1
Desaturation and/or increased FiO2 Unknown	10 (30.3%)	7 (23.3%) 1	0.581
Hypothermia <36.5°C	14 (42.4%)	13 (41.9%)	1
Hypothermia <36°C	6 (18.2%)	4 (12.9%)	0.733

Data are presented as *n* (%).

Tachycardia and bradycardia were defined as heart rate above 180bpm or below 120 bpm respectively for more than 4 s. Desaturation was defined as SpO_2_ below 92% for more than 50 s.

SpO_2_, oxygen saturation; FiO2, fraction of inspired oxygen.

Median blood sugar level was 57 mg/dl. Four neonates presented with a blood sugar level lower than 35 mg/dl, with the lowest recorded at 31 mg/dl. No event of symptomatic hypoglycemia was observed. Blood sugar level assessment was not medically indicated for 7 neonates, therefore were unknown.

Concerning potential technical issues due to the Tandem, no equipment failures leading to the discontinuation of SSC transfers were recorded. No other specific adverse event occurred.

The results of parental and nursing satisfaction with SSC transfer via the Tandem assessed during the study showed a median (min-max) parental satisfaction rate of 10/10 (6/10-10/10) and nurses satisfaction rate of 9/10 (2/10-10/10). Satisfaction rates were unknown for 16 and 11 neonates transferred respectively.

## Discussion

This study is one of the first investigating SSC transfers from the delivery suite or the operating theater to the NICU for both term and preterm neonates requiring admission to the NICU, with or without respiratory support. Prior studies focused on smaller cohorts of neonates, and some of those were in different settings such as ambulance transport (10–12).

No medical adverse events or equipment failures necessitating the discontinuation of SSC transfers via the Tandem were observed. This underscores the technical feasibility of implementing this approach, even in neonates requiring continuous positive airway pressure (CPAP), high-flow nasal cannula (HFNC), intravenous (IV) access, and nasogastric tubes.

The safety of the procedure was evaluated through continuous monitoring of key parameters, including hemodynamic, respiratory, thermal and glycemic parameters, which were recorded at defined time points.

Our results showed that heart rate remained stable and within normal range during the procedure for the majority of term as well as preterm neonates, indicating hemodynamic stability (Figure 3). Furthermore, there was a progressive decrease in heart rate values between setting up SSC and two hours after NICU arrival, suggesting SSC transfers reduced their stress levels overall (Figure 3). Tachycardia (HR > 180bpm) during the procedure was recorded in 12.7% of neonates overall, with the majority solely during setting up SSC. These findings align with studies showing that the heart rate of newborns after birth takes up to an hour to stabilize due to many physiological changes happening during that time to transition from a fetal circulation to a newborn circulation (13).

With or without respiratory support, the median oxygen saturation consistently remained above 95% with a median FiO2 at 21% during the whole procedure for all neonates and in preterm neonates as well, consistent with local protocols and established recommendations (Figure 3) (13–15). Transient desaturations (SpO_2_ < 92%) were recorded in 17 (27%) neonates, with no significant difference in occurrence between the term and preterm groups (Table 4). These desaturation episodes were therefore self-corrected, without any negative clinical outcome. Research has shown that SpO_2_ levels below 92% can manifest under physiological conditions as well, even within healthy term and preterm neonates, due to all the respiratory and circulatory changes that occurs during the early postnatal period (14). Furthermore, Mitchell et al. showed that premature neonates between 27 and 30 weeks of gestational age who received SSC for 2 h a day over 5–10 consecutive days had fewer bradycardia and oxygen desaturation, showing that SSC provided physiological stability and could be more beneficial than standard care in an incubator even during transfers (15).

Neonates are susceptible to heat loss because of their length to body surface ratio. Maintaining an appropriate body temperature in neonates is imperative, as hypothermia can have metabolic repercussions such as hypoglycemia and acidosis and is associated with an increased risk of mortality (9, 16, 17). Although some neonates experienced moderate hypothermia, body temperatures below 36°C were predominantly measured during initial setup of SSC and was attributed mainly to the resuscitation and positioning maneuvers prior to setup. Our study showed that median body temperatures remained consistently within normal range and despite moderate heat loss between departure from the delivery room and the arrival at the NICU, the median temperature increased after admission both in preterm and term neonates. Moreover, the median temperature after 120 min of SSC was higher than during setup, indicating SSC could prevent heat loss overall (Figure 3). We did not compare these findings with a control group transported in an incubator, therefore cannot affirm that SSC is more efficient in preventing heat loss. However, our findings seem to align with studies that showed that heat loss was recorded both during SSC and incubator transfers, calling for additional heat conservation methods by maintaining an acceptable room temperature, stopping air drafts and wearing hats and covers as the main priority regardless of the type of transfer occurring (10, 18). Additionally, our results showed that there was no difference in the occurrence of hypothermia between term and preterm neonates, suggesting that SSC transfers could be applied even in preterm neonates weighing more than 1,500 g.

Glycemic stability is a paramount concern in neonatal care. In our study, only 4 neonates presented with glycemia lower than 35 mg/dl, with the lowest recorded at 31 mg/dl, and none of the neonates developed clinical signs of hypoglycemia. These findings align with guidelines recommending glycemic levels above 35 mg/dl during the early postnatal hours, suggesting that SSC did not exert any adverse effect on blood sugar level (19).

Most of the studies assessing the safety and feasibility of SSC transfers to this day studied small cohorts of term or preterm neonates who did not require respiratory support, and some of those were in different settings such as ambulance transport (10–12).

This present study is one of the first investigating SSC transfer from the delivery room or the operating room to the NICU for not only term but also preterm neonates, including those requiring non-invasive respiratory assistance. The hemodynamic, respiratory and thermal parameters as well as blood sugar level measurements remained stable for the whole procedure. This was comparable to the findings of Hennequin et al. as well as Sontheimer et al, who reported safe SSC ambulance transports (12, 20). These findings were also in line with the study of Van den Berg et al, which compared neonatal ambulance transports with an incubator to SSC transports (11). In this last study, both groups also maintained stable physiological parameters. However, this study was conducted on neonates who did not require respiratory support whereas our study included neonates requiring respiratory support among other medical equipment. Another study by Carneiro et al. compared SSC transfers on fathers to incubator transfers and showed that both transfers induced moderate heat loss (10). Kristoffersen and al. compared moderately preterm infants who received immediate SSC to conventional incubators and showed that early SSC in the delivery room for moderately preterm neonates was feasible and safe as well (21). Our results are in line with studies showing that in high-resource settings, thermal regulation should not be limiting SSC transfers because SSC and incubator transfers maintained the neonate's temperature within normal range (22).

We also aimed to evaluate the parental satisfaction of SSC transfers. The neonates were transported skin-to-skin on their father/mother's birthing partner in 51 (81%) cases, and both parents and nurses reported high levels of satisfaction with SSC transfers (median satisfaction score of 10/10 and 9/10 respectively), indicating that transfers were well appreciated by the parents and the medical staff. These findings were in line with a small study by Lundqvist et al. who conducted open interviews with parents who benefited from SSC ambulance transport, which highlighted the parent's need of feeling important, valued, included and secure in the care of their newborn (23). Sontheimer et al. also showed that parents felt safe and satisfied in those same settings (12).

The strength of our study is that we were able to transfer neonates to the NICU safely using SSC transfers providing zero-separation aligning with the latest WHO recommendations, while implementing continuous monitoring of physiological parameters. However we acknowledge some limitations to our study such as its observational nature and the lack of control group. During the development of the Tandem, our team felt that randomization was not ethical and preferred to provide the possibility of SSC transfers to all participants. Consequently, the lack of a control group transported in standard incubators doesn't allow us to directly compare the two methods. Additionally, the majority of transfers occurred on the father's chest (81%), as neonates often required immediate NICU care while mothers necessitated postpartum attention in the delivery room. Notably, our study played a pivotal role in facilitating a protocol change by allowing maternal postpartum monitoring in close proximity to neonates at the NICU, which enabled an increase in frequency of transfers on the mother's chest. Future investigations should include infants with lower gestational age and birthweight and/or presenting with more severe conditions, to further elucidate the feasibility and safety of all SSC transfers.

## Conclusions

In this study, we investigated the feasibility and safety of implementing skin-to-skin contact for transfers from the delivery room or operating room to the NICU via the Tandem shuttle, for both term and preterm neonates, including those requiring respiratory assistance among other medical equipment. We demonstrated that skin to skin transport for infants >1,500 g using the Tandem is feasible and safe with stable physiological parameters.

Beyond the clinical benefits, SSC transfers emerge as a compelling alternative to conventional incubator transfers, integrating early bonding and parental satisfaction to the immediate postnatal care. Our research paves the way for broader adoption of this approach, underlining its potential to revolutionize neonatal care practices and enhance the well-being of both neonates and their families.

## Data Availability

The raw data supporting the conclusions of this article will be made available by the authors, without undue reservation.
